# A Magnetic Nanoparticle-Based Multiple-Gene Delivery System for Transfection of Porcine Kidney Cells

**DOI:** 10.1371/journal.pone.0102886

**Published:** 2014-07-21

**Authors:** Yan Wang, Haixin Cui, Kui Li, Changjiao Sun, Wei Du, Jinhui Cui, Xiang Zhao, Wenjie Chen

**Affiliations:** 1 Institute of Environment and Sustainable Development in Agriculture, Chinese Academy of Agricultural Sciences, Zhongguancun Haidian District, Beijing, China; 2 Institute of Animal Sciences, Chinese Academy of Agricultural Sciences, Haidian District, Beijing, China; Heidelberg University, Germany

## Abstract

Superparamagnetic nanoparticles are promising candidates for gene delivery into mammalian somatic cells and may be useful for reproductive cloning using the somatic cell nuclear transfer technique. However, limited investigations of their potential applications in animal genetics and breeding, particularly multiple-gene delivery by magnetofection, have been performed. Here, we developed a stable, targetable and convenient system for delivering multiple genes into the nuclei of porcine somatic cells using magnetic Fe_3_O_4_ nanoparticles as gene carriers. After surface modification by polyethylenimine, the spherical magnetic Fe_3_O_4_ nanoparticles showed strong binding affinity for DNA plasmids expressing the genes encoding a green (DNA_GFP_) or red (DNA_DsRed_) fluorescent protein. At weight ratios of DNA_GFP_ or DNA_DsRed_ to magnetic nanoparticles lower than or equal to 10∶1 or 5∶1, respectively, the DNA molecules were completely bound by the magnetic nanoparticles. Atomic force microscopy analyses confirmed binding of the spherical magnetic nanoparticles to stretched DNA strands up to several hundred nanometers in length. As a result, stable and efficient co-expression of GFP and DsRed in porcine kidney PK-15 cells was achieved by magnetofection. The results presented here demonstrate the potential application of magnetic nanoparticles as an attractive delivery system for animal genetics and breeding studies.

## Introduction

Transgenic technology has attracted the attention of scientists in various fields, including medicine [1]–[4], agriculture [5]–[8] and biology [9]–[12]. Gene delivery, the process of introducing foreign DNA into host cells, is a necessary step in the genetic modification of crops and livestock. The bottleneck in the success of genetic transformation has been the development of a safe, stable and efficient gene delivery system [13]. Over the past few decades, many different gene delivery methods have been developed for various types of animal and plant cells. Generally, gene delivery vectors can be divided into viral and non-viral types [14]–[18]. Virus-mediated gene delivery utilizes the ability of a virus to inject its DNA into a host cell. Although viral carriers can achieve relatively high levels of transfection efficiency, they suffer from many disadvantages, including potential risks of toxicity or immunogenicity, difficulty of production scale-up, and limited capacity to carry DNA beyond a certain size [10]. Hence, non-viral carriers, which have advantages such as minimal host immune response, stability in storage, and relative ease of production and scale-up, are being developed as alternative methods of gene delivery [19], [20]. Non-viral approaches include agrobacterium-mediated methods, chemical methods such as lipofection, and physical methods such as microinjection, gene guns, impalefection, hydrostatic pressure, electroporation, continuous infusion, and sonication.

Traditional pronuclear microinjection is the most common non-viral method of exogenous gene delivery into animal cells and is considered the most reliable way to produce transgenic animals. However, in this method, the integration of exogenous genes into the chromosome of the host cell is usually fairly haphazard, resulting in low integration efficiency of the exogenous gene. In addition, pronuclear microinjection requires the use of a large number of animal models to screen for transgenic animals that successfully express the exogenous gene. Therefore, the low efficiency and high cost restrict the application of this method to animal genetic transformation.

Alongside developments in nanotechnology and molecular biology, nanoparticles with positively charged surfaces have shown promise as non-viral carriers for gene delivery [21], [22]. Superparamagnetic nanoparticles have many advantages over other non-viral gene delivery systems, including enhanced resistance to digestion, higher DNA carrying capacity, more powerful penetration, lower cost, and the ability to drive stable and efficient expression of target genes when exposed to an external magnetic field [23]–[26]. Magnetofection is a simple, versatile and highly efficient method of gene transfer that uses magnetic force to promote the uptake of gene vectors associated with cationic magnetic nanoparticles into target cells [27]–[34]. Effective gene delivery into the mammalian somatic cell nucleus is a key step in achieving a reproductive single cell clone and enabling the breeding of new varieties of animals using the somatic cell nuclear transfer technique. Although the application of magnetic nanoparticles as gene carriers has progressed rapidly in many fields, particularly that of gene therapy for human disease [31]–[34], investigations of their potential applications for animal genetics and breeding, especially for multiple-gene delivery, are challenging and only a limited number of studies have been performed.

**Figure 1:**
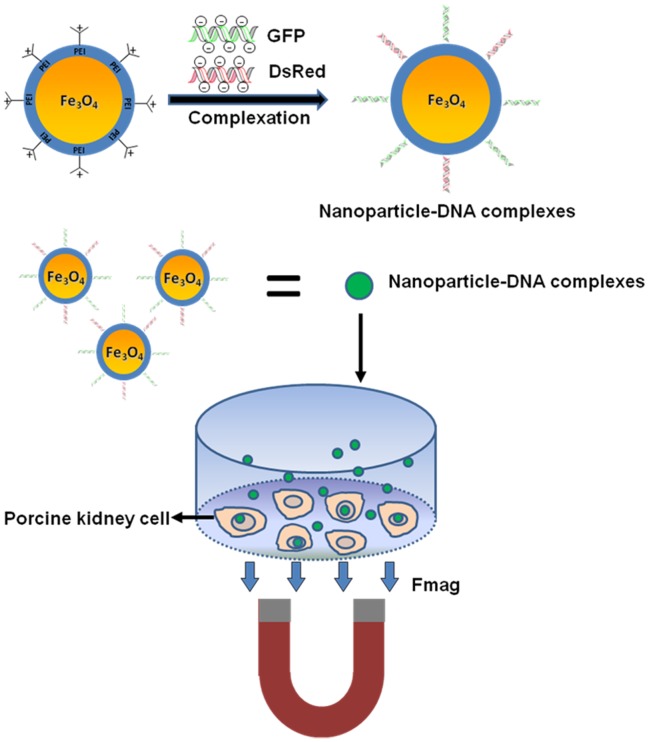
Schematic illustration of the MagNP-based method of multiple-gene delivery into porcine kidney cells. After complexation of plasmids containing the genes encoding GFP and DsRed to the MagNPs, the MagNP-DNA complexes were added to the cells and a magnetic force (Fmag) was applied to promote gene delivery.

Here, polyethylenimine (PEI)-coated superparamagnetic Fe_3_O_4_ nanoparticles (MagNPs) with magnetism and good biocompatibility were used as multiple-gene delivery agents for porcine kidney cells (Figure 1). To enable visualization of the transgenic cells, plasmids containing genes encoding green fluorescent protein (GFP) and a red fluorescent protein (DsRed) were used. Successful co-expression of GFP and DsRed was observed in the transfected cells. To clarify the binding mechanism between the MagNPs and DNA, the microstructure of the MagNP-DNA complexes was investigated. Overall, the gene delivery system described here is simple, low cost, rapid, and capable of delivering multiple genes into mammalian cells. This study provides a new insight into the development of transgenic methods for animals.

## Materials and Methods

### Materials

The MagNPs (1 µg/µl), which were fabricated by surface modification of Fe_3_O_4_ nanoparticles with branched PEI (25 kDa), were purchased from Chemicell (Berlin, Germany). Fetal bovine serum, phosphate-buffered saline and Dulbecco's modified Eagle's medium (DMEM) were purchased from HyClone (Logan, UT, USA). Porcine kidney PK-15 cells were purchased from the National Platform of Experimental Cell Resources for Sci-Tech (Beijing, China). The GFP plasmid, which expressed enhanced GFP under the control of the cytomegalovirus promoter, was purchased from BD Biosciences Clontech (Palo Alto, CA, USA). The DsRed plasmid was obtained from the Harbin Veterinary Research Institute (Chinese Academy of Agricultural Sciences, Harbin, China). The DNA plasmids were expressed in *Escherichia coli* and then isolated and purified using the Vigorous Plasmid Maxprep Kit (Vigorous Biotechnology Beijing Co., Ltd., China), according to the manufacturer's instruction. The plasmid DNA was dissolved in pure water and the purity was confirmed by 1% agarose gel electrophoresis. The DNA concentration was measured by UV absorption at 260 nm using a 2800 UV-vis spectrophotometer (UNIC Shanghai Instruments Co., Ltd., China). The GFP and DsRed plasmids were stored at −20°C until use. The red fluorescent dye 1,1′-dioctadecyl-3,3,3′,3′-tetramethylindocarbocyanine perchlorate (DiI) and the blue fluorescent dye 4′,6-diamidino-2-phenylindole (DAPI) were purchased from Beyotime Institute of Biotechnology (Beyotime, Shanghai, China).

The external magnetic fields are provided by a commercial magnetic neodymium-iron-boron (NdFeB) multiwell plate. (MagnetoFACTOR-24 plate, Chemicell, Berlin, Germany). The strength of the NdFeB permanent magnets is 0.3 Tesla. The polarity is alternated north and south. The MagnetoFACTOR plate its special geometry produces strong magnetic fields under each well of 24-well plates.

### Scanning electron microscopy (SEM) of MagNPs

The morphologies of the MagNPs were examined using a JSM-6700F scanning electron microscope (JEOL, Tokyo, Japan). To prepare the SEM sample, one drop of diluted MagNP solution was placed onto a small tin-foil plate and dried at room temperature.

### Preparation and gel electrophoresis of the MagNP-DNA complexes

The binding capacity of the MagNPs for the DNA plasmids containing the genes encoding GFP and DsRed was determined by agarose gel electrophoresis using the Gel Doc EZ gel imaging system (Bio-Rad Laboratories, Inc., Hercules, CA, USA). The gels contained 1% (w/v) agarose in 100 ml of TAE buffer (200 mM Tris, 200 mM acetic acid, and 5 mM EDTA) containing 3 µl of ethidium bromide (0.5 µg/ml) as a stain. To prepare the MagNP-DNA complexes for single gene expression, 1 µg of the GFP or DsRed plasmid was used. For multiple-gene expression, a solution containing 0.4 µg of the GFP plasmid and 0.6 µg of the DsRed plasmid was used. Complexes with DNA:MagNP weight ratios of 0.1∶1 to 20∶1 were prepared at pH 7.4. After incubation at room temperature for 30 min to allow complex formation, the samples were electrophoresed in a 1% (w/v) agarose gel for 30 min at 90 V.

### Atomic force microscopy (AFM)

A Multimode NS-3a atomic force microscope (Veeco, Santa Barbara, CA, USA) was used to examine the morphology and microstructure of the MagNPs and MagNP-DNA complexes. The AFM samples were prepared by mixing plasmid DNA with MagNPs, incubating the mixtures for 30 min, dropping the samples onto fresh sheets of glass, and then air-drying.

### Particle size and zeta-potential measurements

The size distribution and zeta-potential of the MagNP-DNA complexes in deionized water were evaluated using a ZetaPALS analyzer (Brookhaven Instruments Corporation, Holtsville, NY, USA). All measurements were performed in cuvettes. The average particle size was expressed as the volume mean diameter.

### In vitro magnetofection

The DNA plasmids were diluted in serum-free DMEM medium to get a final concentration of 0.005 µg/µl. For multiple-gene expression, the weight ratio of the GFP plasmids to the DsRed plasmids was fixed at 2∶3. Add the 200 µl diluted DNA solution to 1 µl MagNP solution (1 µg/µl) and mix immediately by vigorous pipetting, and then incubated for 30 min at room temperature to form MagNP-DNA complexes and achieve the efficient binding of DNA to the magnetic particles.

PK-15 cells were seeded into a 24-well plate, cultured in DMEM containing 10% fetal bovine serum, and grown to 70–80% confluence. Prior to transfection,the medium was removed and the cells were washed once with phosphate-buffered saline, and then the medium was replaced with fresh serum-free medium. The prepared 200 µl/well MagNP-DNA complexes solutions were added to the 24-well cell culture plate. After mixing, the cell culture 24-well plate was placed on top of a MagnetoFACTOR-24 plate for 6 h incubation at 37°C in an atmosphere containing 5% CO_2_, which ensures equal application of the magnetic field under each well.

After transfection, the medium was replaced with fresh serum-containing medium and the cells were cultured for a further 24 h. To investigate localization of the exogenous GFP after magnetofection, the cells were labeled with the membrane-specific red fluorescent dye DiI and the nucleus-specific blue fluorescent dye DAPI.

### Evaluation of gene expression by confocal microscopy

Co-expression of GFP and DsRed was evaluated by confocal laser scanning microscopy (A1R-Si, Nikon, Yokohama, Japan). The transfected PK-15 cells were plated into Petri dishes for further measurements.

### Flow cytometry

GFP- and DsRed-transfected cells in growth medium were centrifuged and then washed twice with phosphate-buffered saline. The total green (GFP) and red (DsRed) fluorescence intensities and the percentages of transfected cells were determined using a FACSCalibur flow cytometer (BD Biosciences, San Jose, CA, USA).

## Results

### SEM analyses of the PEI-coated MagNPs

**Figure 2:**
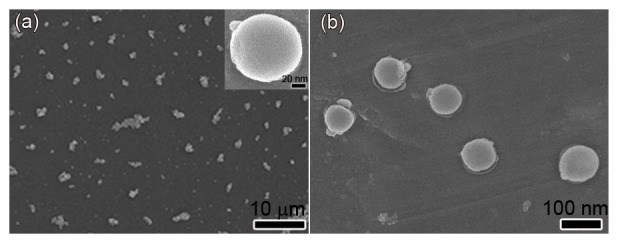
SEM images of PEI-coated Fe_3_O_4_ nanoparticles. (a,b) The high concentration (50 µg/ml) sample of the PEI-coated MagNPs showing slight aggregation of the nanoparticles. The inset shows a single spherical nanoparticle. (b) Higher magnification of the low concentration (10 µg/ml) sample of the PEI-coated MagNPs.

SEM was used to examine the morphology of two different samples of the PEI-coated Fe_3_O_4_ MagNPs. Compared with those in the low concentration sample (10 µg/ml), the MagNPs in the high concentration (50 µg/ml) sample had a larger average size and displayed slight aggregation (Figures 2a and 2b), which occurred as a result of the highly concentrated PEI on the surface that caused conglutination of individual nanoparticles. As shown in the inset in Figure 2a, the MagNPs in both the low and high concentration samples formed spherical structures. The average diameter of the PEI-coated MagNPs was approximately 100 nm (Figure 2b). These results indicate that the concentration of the modified MagNPs should be lower than 10 µg/ml to avoid conglutination.

### The DNA binding capacity of the MagNPs

**Figure 3:**
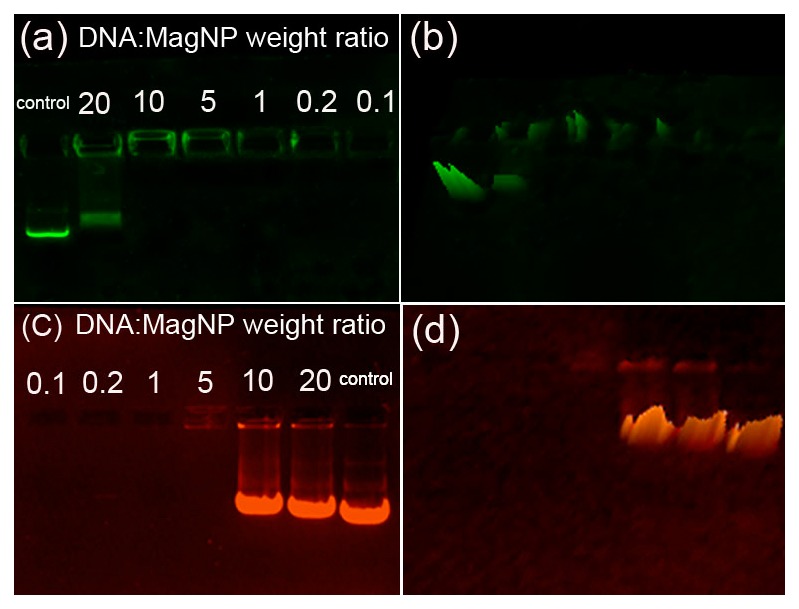
Agarose gel electrophoresis of MagNP-DNA complexes with different DNA:MagNP weight ratios. (a) Migration of MagNP-DNA_GFP_ complexes with DNA:MagNP weight ratios of 20∶1, 10∶1, 5∶1, 1∶1, 0.2∶1, and 0.1∶1. (b) Corresponding 3D projection of Figure 3a. (c) Migration of MagNP-DNA_DsRed_ complexes with the same DNA:MagNP weight ratios. (d) Corresponding 3D projection of Figure 3c. Pure DNA plasmids were used in each case as controls.

Agarose gel electrophoresis was used to determine the binding capacity of the MagNPs for plasmid DNA. Two plasmids containing the gene encoding a green (GFP; DNA_GFP_) or red (DsRed; DNA_DsRed_) fluorescent protein were used as markers. The weight ratios of the GFP and DsRed plasmids to the MagNPs were fixed at 20∶1, 10∶1, 5∶1, 1∶1, 0.2∶1, and 0.1∶1. Migration of DNA_GFP_ in the gel was retarded at DNA:MagNP ratios of 10∶1 and lower (Figures 3a and 3b), and migration of DNA_DsRed_ was retarded at ratios of 5∶1 and lower (Figures 3c and 3d), indicating the formation of MagNP-DNA complexes at these ratios. These results suggest that the binding affinity of the MagNPs for the GFP plasmid was stronger than that for the DsRed plasmid.

### The mechanism of binding of DNA plasmids to the MagNPs

**Figure 4:**
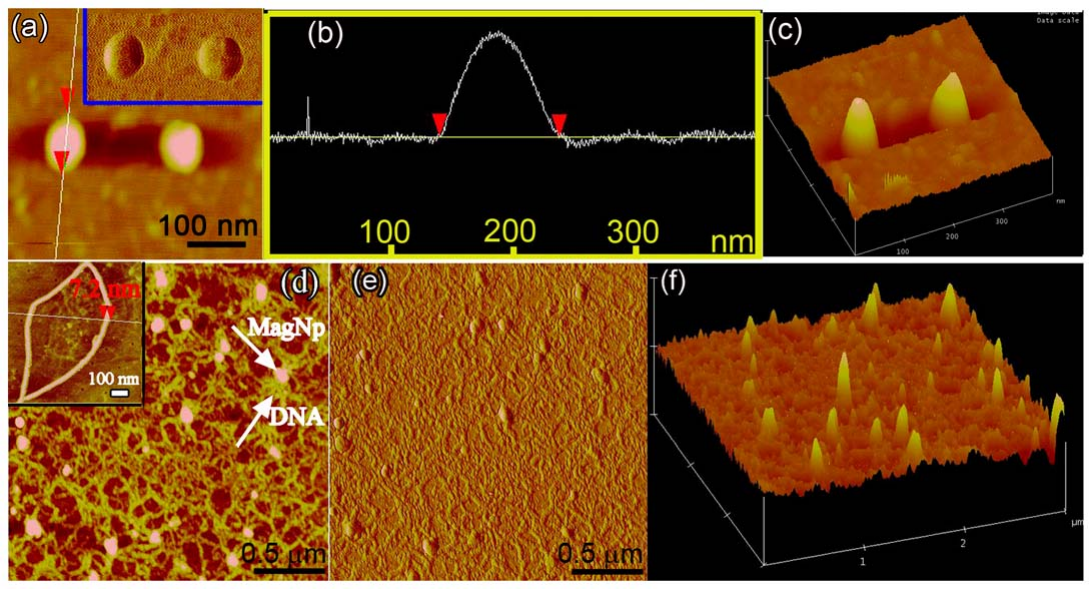
AFM images of MagNPs and MagNP-DNA complexes. (a–c) AFM images of MagNPs. (a) The red arrowheads indicate the diameter of the MagNPs and the inset shows the corresponding phase image. (b) The topographic distance profile corresponding to the region between the red arrowheads in Figure 4a. (c) 3D rendering of the individual MagNPs shown in Figure 4a. (d–f) AFM images of MagNP-DNA complexes. (d) MagNPs are bound to the stretched DNA strands. The local and representative DNA strands are shown in the inset. The red arrowheads indicate the height of the DNA strands. (e) Phase image of MagNP-DNA complexes. (f) 3D rendering of the MagNP-DNA complexes shown in Figure 4d.

To understand the mechanism of binding of DNA to the MagNPs, AFM was used to investigate the microstructures of the MagNPs and MagNP-DNA complexes. Figures 4a–c show representative three-dimensional (3D) topographies of the nanoparticles. As shown in the inset of Figure 4a, the MagNPs were individual spherical nanoparticles with uniform structures. The AFM analysis confirmed that the diameter of a single MagNP was approximately 100 nm, which correlated with the SEM results. The 3D nature of the nanoparticles on the substrate was clearly observable (Figure 4c) and the nanoparticles appeared to be protuberant.

Figures 4d–f show representative AFM images of MagNP-DNA complexes. Several spherical MagNPs were bound to the stretched DNA strands in a net-like structure. Stretched DNA strands of several hundred nanometers in length and 7.2 nm in height were observed on the MagNPs, indicating that they consisted of a bunch of connected pieces of single DNA strands. The DNA bunches remained joined and formed loops of up to several hundred nanometers in length. The corresponding 3D image of the MagNP-DNA complex (Figure 4f) indicated that the surface of the MagNP-DNA complex was not smooth due to the DNA strands attracted on the surface of MagNPs, confirming the formation of the complexes. Formation of a MagNP-DNA complex favors the protection of DNA strands from nuclease degradation. These AFM results confirmed that the attractive interaction between MagNPs and DNA led to the formation of MagNP-DNA complexes.

### Size distribution and zeta-potential measurements

**Figure 5:**
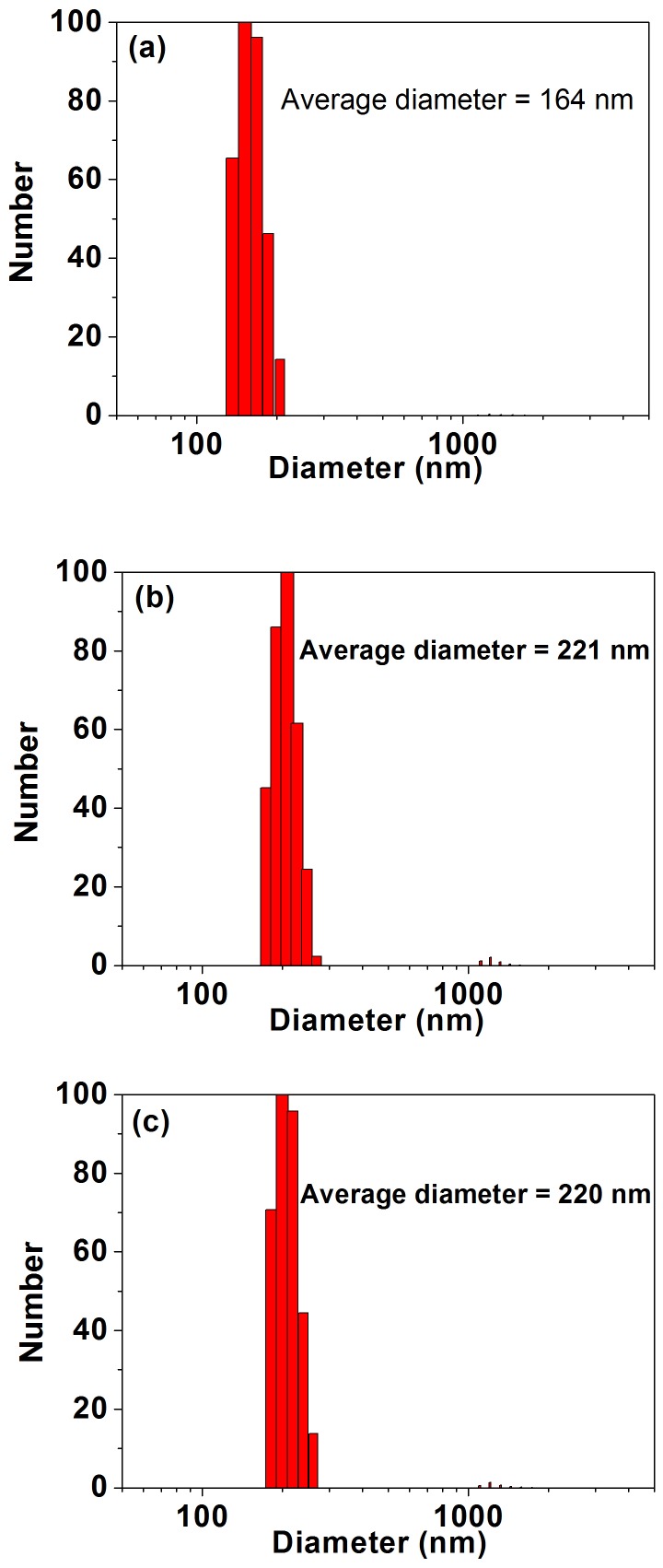
Histograms of the particle size distribution of MagNPs and MagNP-DNA complexes. (a) MagNPs. (b) MagNP-DNA_GFP_ complexes. (c) MagNP-DNA_DsRed_ complexes.

The average size (diameter) of the MagNPs determined using a ZetaPALS particle size analyzer was 164 nm (Figure 5a), which is larger than that determined by the SEM analysis (Figure 2). This discrepancy can be explained by the higher concentration of sample solution used in the ZetaPALS analysis and the formation of a hydrolyzed layer on the particle surface. As expected, the MagNP-DNA_GFP_ and MagNP-DNA_DsRed_ complexes were larger than the individual MagNPs, with mean particle sizes of 221 nm and 220 nm, respectively. The surfaces of the as-prepared MagNP-DNA_GFP_ and MagNP-DNA_DsRed_ complexes were positively charged, with zeta-potential values of +31.09 mV and +48.03 mV, respectively.

### Gene transfection in vitro

**Figure 6:**
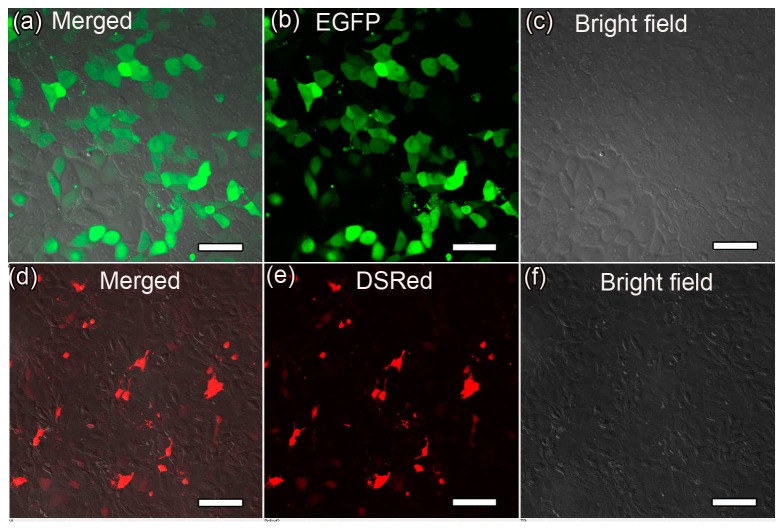
Fluorescence microscopy images of GFP and DsRed expression in transfected PK-15 cells. Green or red fluorescence was detected 24-DNA_GFP_ (a–c) or MagNP-DNA_DsRed_ (d–f) complexes, respectively. (Scale bars, 100 µm).

**Figure 7:**
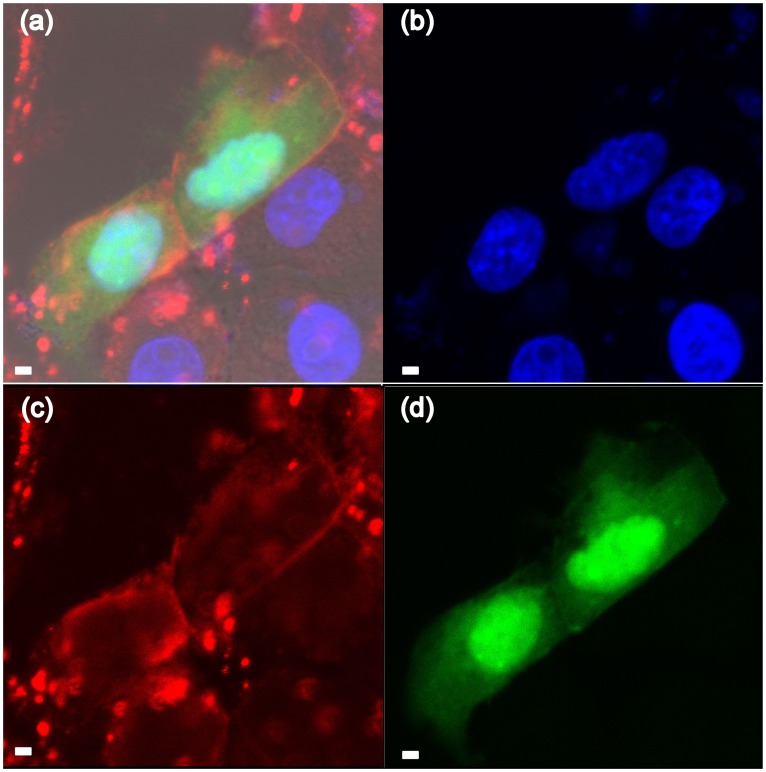
Fluorescence microscopy analyses of the localization of exogenous GFP in transfected PK-15 cells. PK-15 cells magnofected with the MagNP-DNA_GFP_ complex were stained with a membrane-specific red fluorescent dye (DiI) and a nucleus-specific blue fluorescent dye (DAPI) 24 h after transfection. (a) Merged image showing the red membrane, blue nucleus, and GFP (green) expression. (b) DAPI staining only. (c) DiI staining only. (d) GFP signal only. (Scale bars, 20 µm).

First, single expression of GFP or DsRed in porcine kidney PK-15 cells was performed using the MagNPs as gene carriers under a magnetic field. GFP fluorescence was detected 24 h after magnofection of the cells (Figures 6a–c) and the expression efficiency reached 30.5%. DsRed fluorescence was also observed at the same time-point but was not as intense as the GFP signal (Figures 6d–f), which may be explained by unstable binding of the DsRed plasmid to the MagNPs. To examine the localization of the exogenous GFP, the cells transfected with the MagNP-DNA_GFP_ complex were stained with a membrane-specific red fluorescent dye DiI and a nucleus-specific blue fluorescent dye DAPI. The GFP signal was detected in the region located between the red-stained membrane and the blue-stained nucleus (Figure 7a), as well as in the nucleus itself, indicating that the MagNPs were able to deliver the exogenous gene into the PK-15 cells and permit expression in the nucleus.

**Figure 8:**
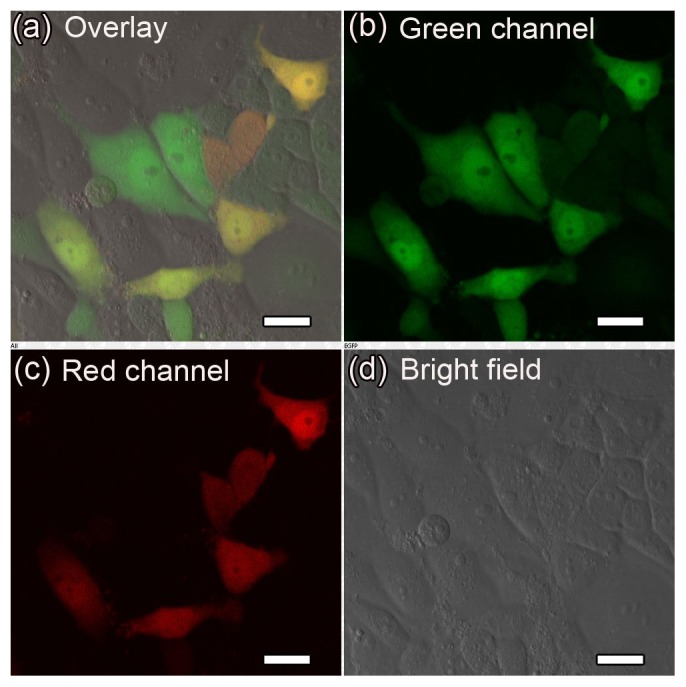
Fluorescence microscopy analyses of co-expressed GFP and DsRed in transfected PK-15 cells. PK-15 cells were co-magnofected with the MagNP-DNA_GFP_ and MagNP-DNA_DsRed_ complexes and images were collected 24 h after transfection. (a–d) Fluorescence (a–c) and bright field imaging (d) of the cells spread between two glass cover slips. GFP and DsRed fluorescence were detected in the green (500–530 nm) and red (552–617 nm) channels, respectively. (Scale bars, 20 µm).

**Figure 9:**
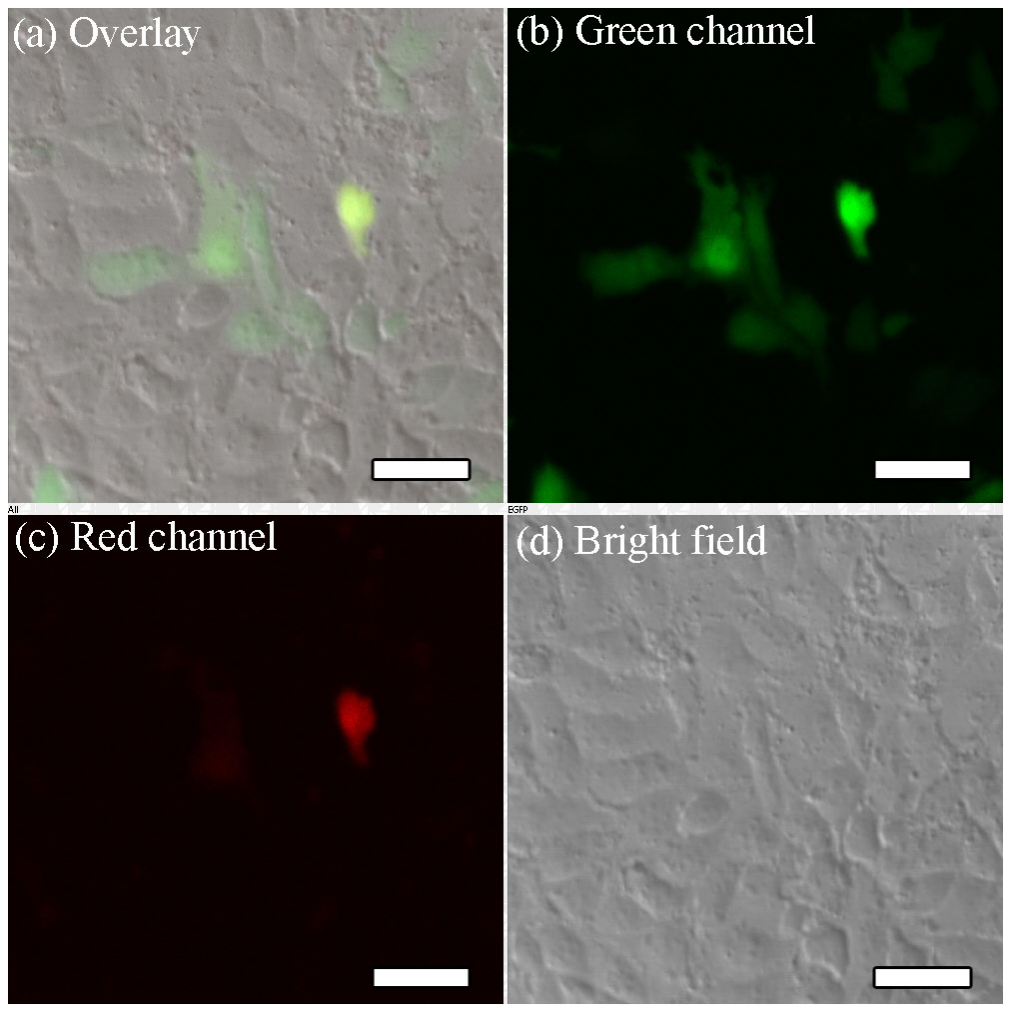
Fluorescence microscopy analyses of GFP and DsRed in PK-15 cells co-transfected without a magnetic field. PK-15 cells were co-transfected with the MagNP-DNA_GFP_ and MagNP-DNA_DsRed_ complexes in the absence of an external magnetic field and images were collected 24 h after transfection. (a–d) Fluorescence (a–c) and bright field imaging (d) of the cells spread between two glass cover slips. GFP and DsRed fluorescence were detected in the green (500–530 nm) and red (552–617 nm) channels, respectively. (Scale bars, 50 µm).

Next, we examined the levels of GFP and DsRed fluorescence 24 h after co-transfection of PK-15 cells with the MagNP-DNA_GFP_ and MagNP-DNA_DsRed_ complexes under a magnetic field. Co-expression of the green and red fluorescent proteins was demonstrated by the detection of yellow fluorescence in the cells (Figure 8a). Control experiments were also performed without external magnetic fields; as expected, the efficiency of co-expression of GFP and DsRed in transfected PK-15 cells was reduced markedly in the absence of the magnetic field (Figure 9).

### Efficiency of co-expression of GFP and DsRed in PK-15 cells

**Figure 10:**
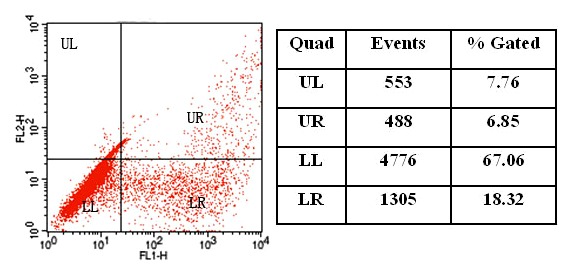
Flow cytometry analysis of the co-expression of GFP and DsRed in transfected PK-15 cells. Cells expressing GFP, DsRed, and GFP plus DsRed are shown in the lower right (LR), upper left (UL), and upper right (UR) quadrants, respectively. The percentages of cells in each quadrant are shown in the table on the right.

To quantitatively study the expression of GFP and DsRed in PK-15 cells co-transfected with the MagNP-DNA complexes under a magnetic field, the cells were examined by flow cytometry. The cell suspension prepared from the co-transfected cells contained 6.85% double-positive cells. Approximately 18.32% of the cells expressed GFP alone and approximately 7.76% expressed DsRed alone (Figure 10).

## Discussion

The development of efficient gene carriers is an essential prerequisite for successful gene delivery. Key issues for successful gene delivery are safety, bioavailability, reproducibility, targeting, capacity, and stability [35]–[38]. This study describes a gene delivery system that addresses these issues by using superparamagnetic Fe_3_O_4_ nanoparticles as carriers for multiple-gene transfer. Superparamagnetic Fe_3_O_4_ nanoparticles modified with positively charged PEI polymers form strong electrostatic interactions with negatively charged DNA to generate MagNP-DNA complexes. Formation of these complexes protects the DNA against nuclease degradation after introduction into cells. The superparamagnetic component enables the MagNP-DNA complex to respond to an external magnetic force, which speeds up the targeting and sedimentation of the gene on the cell surface and reduces the duration of the transfection procedure, resulting in increased cellular endocytosis of the DNA and higher efficiency of exogenous gene expression.

Particle size is an important determinant of cellular uptake. Polycation-DNA gene delivery systems mostly enter the cell by endocytosis or pinocytosis, resulting in a size limitation for maximum endocytosis [39], [40]. Cellular uptake of inorganic particles, for example through constitutive endocytosis, is also affected by the particle size [41], [42]. The SEM analysis performed here demonstrated that the MagNPs were spherical in shape with an average diameter of approximately 100 nm, enabling them to enter the cell nucleus much more easily than bulky materials. Hence, magnetic nanoparticles are favorable for carrying DNA molecules into cells because of not only the absorption effect promoted by the application of an external magnetic field, but also their accelerated cellular intake, which circumvents degradation of the complexes by nucleases and is crucial for optimal gene delivery.

Dispersity is another key factor that determines the performance of gene carriers. The SEM analyses showed that the concentration of MagNPs should be kept reasonably low for good dispersity of the particles in solution. As a result of conglutination caused by the PEI molecules, aggregates were formed when the concentration of MagNPs was higher than the suitable range. Surface charge and size of MagNP-DNA complexes are also important determinants of cellular uptake. Complexes with positively charged surfaces form electrostatic interactions with negatively charged cellular membranes [40]. The mean particle sizes of the MagNP-DNA_GFP_ and MagNP-DNA_DsRed_ complexes were 221 nm and 220 nm, respectively. These complexes had positively charged surfaces with zeta potentials of +31.09 mV and +48.03 mV, respectively. The agarose gel electrophoresis analysis confirmed that the higher surface charge of the MagNP-DNA_DsRed_ complex compared with the MagNP-DNA_GFP_ complex resulted from a lower binding affinity of the MagNPs for the DsRed plasmid than the GFP plasmid.

The gel retardation analysis demonstrated a strong interaction between the plasmid DNA and the MagNPs. The DNA molecules were unable to migrate and remained in the loading wells of the gel when the DNA:MagNP weight ratio was lower than or equal to 10∶1 (for DNA_GFP_) or 5∶1 (for DNA_DsRed_). Kim et al. [43] investigated the use of structurally diverse arginine-conjugated PAMAM dendrimers as gene delivery systems and showed that all of the polymers examined retarded DNA completely at polymer:DNA ratios higher than 2∶1. Bardi et al. [44] investigated the biocompatibility and gene carrying performance of amino-functionalized CdSe/ZnS quantum-dot-doped SiO_2_ nanoparticles and demonstrated that DNA can be carried by these nanoparticles at a ratio of 2.5 ng of DNA/mg of 50 nm nanoparticles, or 5 ng of DNA/mg of 25 nm nanoparticles. Here, 5–10 ng of DNA could be delivered into PK-15 cells by only 1 ng of the MagNPs, indicating that the MagNPs used in this study have strong binding affinities for DNA molecules. For efficient gene delivery, it is important that MagNPs can bind to plasmid DNA and form stable complexes; adequate binding of MagNPs to DNA ensures protection of the DNA against degradation in vivo and prevents dissociation of the MagNP-DNA complexes.

The mechanism of binding of DNA to MagNPs is worth investigating. Recent studies have suggested that DNA molecules can be condensed by a gene carrier until they reach the nucleus and compacted into a size that is smaller than the nuclear pores [45], [46]. However, the spherical MagNPs used here bound to a number of stretched DNA strands up to several hundred nanometers in length. The MagNPs appeared to be enclosed by a large number of net-like DNA bunches consisting of connected pieces of single DNA strands, thereby enabling them to carry large amounts of DNA into the PK-15 cells. Therefore, efficient sedimentation of MagNP-DNA complexes in the presence of an external magnetic field ensures that a minimal DNA dose is sufficient to achieve a high level of transfection efficiency.

Here, magnetofection was used as a novel method for the transfection of pig somatic cells [28], [47]. Two processes are involved in this method: the association of carriers with superparamagnetic Fe_3_O_4_ nanoparticles, and gene delivery under the application of a magnetic field. Conventional gene delivery methods have been used widely in agriculture. For example, gene guns have been used to deliver DNA-coated gold particles, which can be bombarded directly into the cytoplasm and nuclei of cells, to facilitate the expression of target genes [48]. However, the disadvantage of gene gun bombardments is that the non-biodegradable gold particles may cause adverse side effects when they accumulate in cells [49], [50]. The results presented here demonstrate that the use of PEI-modified superparamagnetic Fe_3_O_4_ nanoparticles as gene carriers enables reproducible, rapid and efficient transfection of PK-15 cells. This method simplifies the transfection process and can achieve simultaneous expression of multiple genes in porcine somatic cells.

High levels of fluorescence were observed 24 h after delivery of DNA_GFP_ or DNA_DsRed_ into PK-15 cells using the MagNPs as carriers. Compared with DsRed, the expression of GFP was stronger and the expression efficiency reached 30.5%, which can be attributed to the relatively stronger binding affinity of the MagNPs for this DNA plasmid. Further analysis of the localization of the GFP signal in the transfected cells revealed that the gene was expressed in the nucleus. Endonuclear expression of exogenous genes in mammalian somatic cells is an important step forward for reproductive cloning using the somatic cell nuclear transfer technique; therefore, magnetic nanoparticles may be useful for carrying genes into mammalian somatic cells for animal breeding and genetic modifications.

Simultaneous expression of GFP and DsRed in PK-15 cells was also achieved using the PEI-modified MagNPs as gene carriers under external magnetic fields. Application of the external magnetic field was important for efficient co-expression, which was reduced markedly in the absence of this force. The flow cytometry analysis showed that 6.85% of the cells co-transfected with the two fluorescent proteins were double-positive, which was lower than the percentages of co-transfected cells expressing GFP alone or DsRed alone (18.32% or 7.76%, respectively). These results indicate that the expression efficiency of GFP was higher than that of DsRed. Cationic liposomes are frequently used gene carriers that provide high transfection efficiency and high levels of transgene expression [51], [52]. Although cationic liposome systems can deliver multiple genes into cells, they are hampered by poor reproducibility and low co-transfection efficiency. By contrast, the results presented here show that stable simultaneous expression of GFP and DsRed in PK-15 cells can be achieved using MagNPs as gene carriers. This gene delivery system has great potential for magnetofection for somatic cell nuclear transfer. Future studies will focus on optimizing the formulation for higher transfection efficiency and evaluating the performance of the MagNPs as a gene delivery system in vivo.

## Conclusion

We have developed a rapid and stable co-expression system for delivering multiple genes into the porcine somatic cell nucleus by magnetofection. PEI-modified Fe_3_O_4_ magnetic nanoparticles have a strong binding affinity for DNA and excellent biocompatibility. The small size of magnetic nanoparticles facilitates their effective binding to DNA and the successful transfer of exogenous plasmids into mammalian cells. The spherical MagNPs bound to several stretched DNA strands with lengths of up to several hundred nanometers. This gene delivery system is simple, low cost, and rapid. The successful demonstration of multiple-gene delivery into the mammalian somatic cell nucleus represents an important step forward for reproductive cloning using the somatic cell nuclear transfer technique.
